# A suitable anaesthetic protocol for metamorphic zebrafish

**DOI:** 10.1371/journal.pone.0246504

**Published:** 2021-03-05

**Authors:** Jennifer P. Owen, Robert N. Kelsh

**Affiliations:** Department of Biology and Biochemistry, University of Bath, Bath, United Kingdom; University Zürich, SWITZERLAND

## Abstract

Zebrafish are frequently used as a means to investigate development. These studies increasingly require repeated anaesthesia of zebrafish during juvenile (i.e. metamorphic) stages. The effects of anaesthesia during this time remain poorly studied. The aim of this study was to develop a reliable method that can be used for frequently repeated anaesthesia during juvenile stages. Initially, we assessed different concentrations of MS-222, the most commonly used fish anaesthetic, for 30 minute anaesthesia with recovery. We showed that suitable MS-222 doses could be identified for the smallest (7mm) and largest (20mm) fish. However, we found that juvenile fish within a specific metamorphic window (sized between 8–16 mm) were vulnerable to MS-222 and no standard concentration of MS-222 provided reliable anaesthesia under these conditions. Hence we focussed our efforts on identifying a protocol for these stages. We tested six different published anaesthesia protocols P1—P6 where P1, P2 corresponds to 0.01% MS-222, P3, P4: 0.085% 2-phenoxyethanol and P5, P6: 0.00025%/0.0050% Propofol/Lidocaine. In protocols P1, P3, P5 fish were maintained by immersion, whilst in P2, P4 and P6: fish were maintained on an anaesthetic-doused cotton-pad. We assessed reliable anaesthesia using 10 fish for 10 minutes, with full recovery. Our data allowed us to eliminate two of these protocols as unsuitable for short term anaesthesia with recovery of juvenile fish. Extending these studies to explore repeated anaesthesia at 4 day intervals for 20 days under the remaining four protocols, we showed that P1 and P4 were both suitable for repeated anaesthesia, and that P4 was most suitable for imaging. We confirmed that P4 remained suitable when the frequency of anaesthesia was increased to every 2 days. We conclude that this protocol provides a refinement to the current protocol for repeated anaesthesia with recovery of juvenile zebrafish in the vulnerable metamorphic window.

## 1 Introduction

Laboratory zebrafish (*Danio rerio*) were originally developed as a model organism for developmental biology, but their use has since spread to incorporate congenital and degenerative diseases, regeneration, and toxicology [1,2]. For example, within developmental biology, zebrafish have become a key model organism for the understanding of pigment pattern formation [3,4]. Zebrafish generate striking horizontal blue and yellow stripes during the metamorphic period between 21–70 days post fertilisation (dpf) which is caused by the self-organisation of pigment producing cell types across the body of the skin [5]. Many investigations have been undertaken in the last decade in order to understand the cell and molecular biology underpinning the self-organisation of these cells during this time [5–8].

Central to documenting the development of the pigment pattern is repeated photography of the pigment cell distribution in juvenile fish throughout this period; these images can then be used to track the appearance, migration and death of cells on an individual zebrafish during this period. Photography requires general anaesthesia of the fish, and this needs to be repeatedly applied to document the progression of pattern formation. Other general non-invasive techniques that may require repeated anaesthesia over a short period of time include imaging of developmental processes [9], mucus collection and other non-lethal sampling methods [10,11].

General anaesthesia is defined as a temporary loss of sensation and awareness through depression of the central nervous system. This state may be followed by different levels of analgesia (absence of pain) and muscle relaxation. The use of an appropriate anaesthetic protocol for scientific procedures is important. Fish that are not anaesthetised properly may experience stress and pain during the procedure compromising animal welfare. Poor anaesthesia can also lead to data variability; for example, fish that move during imaging can impede imaging. On the other hand, fish that are anaesthetised too deeply may not recover, cutting time-series studies short.

To date, a limited number of anaesthetics have been rigorously tested, with almost all work focusing on the very young (embryonic/early larval, <5 dpf [12]) and adult (>100 dpf) [13–17] fish, with juvenile stages being neglected. MS-222 (tricaine methanesulfonate) is the anaesthetic that has been the most largely used by the scientific community [18]. MS-222 is a muscle relaxant that blocks sodium and to a lesser degree potassium currents in nerve membranes [19]. It is a water-soluble anaesthetic commonly used for fishes and other cold-blooded animals and is considered safe [17,20,21]. However, recent studies have suggested that MS-222 has limitations. Without the correct dose and exposure time, MS-222 in adult zebrafish can have adverse side effects such as aversion and stress induction [16], epidermal and corneal lesions, hypoxemia, decreased heart rate and higher mortality under long-term sedation [22]. Such effects can be an issue when deeper stages of anaesthesia and long duration procedures are needed, as well as when repeated imaging is required [12].

Aside from MS-222, a limited number of other anaesthetics have also been used or tested on adult zebrafish. These include; 2-phenoxyethanol [14], etomidate [23], propofol [23,24], lidocaine [23,24] and ketamine [23], although so far there is no conclusive evidence to suggest that these compounds are better than MS-222 itself. Other alternatives consist of combinations of these compounds. For example, in comparison with using MS-222 alone, low doses of isoflurane with MS-222 have been shown to prolong the length of safe anaesthesia (anaesthesia duration without death) and to increase the speed of recovery in adult zebrafish, with minimal effects on the heart rate [22]. More recently, Valentim *et al*, trialled various combinations of analgesics with anaesthetics, of which they found that propofol with lidocaine was the most successful combination in anaesthetising and recovering adult fish [23,24]. They found that there were various advantages and disadvantages when comparing the combination with MS-222 alone and advocated tailoring anaesthetic regime to experimental requirements [24].

In summary, whilst there has been an increase in the use of juvenile zebrafish in developmental studies, there is no rigorously tested protocol for anaesthesia in juvenile stages. Furthermore, whilst MS-222 has been identified as a reasonable anaesthetising agent in adult fish [13,17,18], its adverse effects leave uncertainty as to whether it is the most refined option for juvenile fish. In the light of new alternatives, it is possible that other treatments are more suitable at this sensitive developmental stage, especially in the context of repeated treatment.

In this three-part study, we determine an anaesthetic protocol for repeatedly anaesthetising and recovering juvenile fish. Firstly, we investigate the effects of different concentrations of MS-222 as suitable for single-use anaesthesia with recovery for juvenile fish. From this investigation, we demonstrate a juvenile developmental time window within which zebrafish are particularly sensitive to MS-222. Because fish of intermediate length were highly sensitive to the concentration of MS222, it is difficult to define appropriate standardised procedures for anaesthesia during this developmental stage. Secondly, we focus on this sensitive time window, using a shorter anaesthesia window (10 mins), broadening our range of anaesthetics, and trialling different methods for maintenance of anaesthesia, to determine an effective anaesthesia protocol for permitting a workable duration of anaesthesia with good recovery. In particular alongside 0.01% MS-222, we test a combination of 0.00025%/0.0050% propofol and lidocaine. We choose a concentration of propofol with lidocaine identified as the most successful combination in anaesthetising and recovering adult fish [23,24]. We chose 2-phenoxyethanol due to its rapid action and fast, uneventful recovery in other fish [25].

Next we determined a protocol for repeated anaesthesia during the juvenile stage. We used the four most promising protocols from experiment B in a repeated anaesthetic trial and monitored the fish for any developmental limitations or reduced survival. We showed that of these four protocols, a combination of 0.085% 2-phenoxyethanol with maintenance by cotton pad was the most suitable protocol for imaging due to low breath rate, although a concentration of 0.01% MS-222 with maintenance by immersion was also suitable. Finally, we repeat the experiment using the most successful protocol increasing the frequency of the anaesthesia to every 2 days instead of every 4 days for 10 fish. This experiment too meets our criteria for success, therefore we propose that this protocol (2-phenoxyethanol at a concentration of 0.085% maintained using a cotton pad doused in anaesthetic) provides the best currently available protocol for repeated anaesthesia with recovery during the 8–16 mm Standard Length (SL) sensitive stages.

## 2 Materials and methods

### 2.1 Ethics statement

This study was performed with the approval of the University of Bath ethics committee and in full accordance with the Animals (Scientific Procedures) Act 1986, under Home Office Project Licenses 30/2937 and P87C67227.

### 2.2 Facility conditions

#### 2.2.1 Fish housing

All fish were wild-type AB strain, bred in-house for >20 years. The fish were housed according to FELASA recommendations [26], in tanks filled with circulating system water at 28±0.2oC. System water is made up from Reverse Osmosis water, with synthetic sea salt added at an amount such that the conductivity of the water is kept at approximately 800uS. Average water quality data are as follows: pH: 7.30, general Hardness: 160mg/l CaCO3, Ammonia: 0 mg/l NH3, Nitrite: 0 mg/l NO2, Nitrate: < 10 mg/l NO3- and Conductivity: around 800μS/cm. Light cycle is 14 hours light/10 hours dark (Lights on at 08:00, off at 22:00 daily). All fish were in general good health and no specific diseases were observed throughout the colony. Prior to the experiments and in experiment A, fish were housed in groups of no more than 30 fish. From the start of experiments B and C, fish were housed in groups of 10. From the start of experiment D, fish were housed individually.

#### 2.2.2 Feeding

The fish were fed Paramecium from 5-15dpf, followed by Ziegler Larval AP100 powder from day 16 – 22dpf. From 23dpf onwards they are fed Sparos Zebrafeed and Brine Shrimp.

#### 2.2.3 Schedule 1 killing

Fish euthanized by schedule 1 killing were given an overdose of an anaesthetic (0.2% MS-222) followed by crushing of head to ensure death.

### 2.3 Anaesthetic preparation

#### 2.3.1 MS-222 preparation

A 0.4% buffered MS-222 stock solution was prepared by dissolving ethyl-2-amino-benzoate methanesulfonate powder (Sigma-Aldrich) in system water from the Bath Zebrafish Facility, and the pH adjusted to 7.0 using sodium bicarbonate. Working dilutions were prepared by diluting this stock solution with sterile water.

#### 2.3.2 2-phenoxyethanol preparation

A working solution of 0.085% 2-phenoxyethanol was prepared by diluting 2-phenoxyethanol (Sigma-Aldrich) with system water.

#### 2.3.3 Propofol/lidocaine preparation

A working solution of 0.00025%/0.0050% Propofol/lidocaine mix was prepared by dilution of 1% propofol (Lipuro 1%, B. Braun Melsungen AG, Germany) and 1% lidocaine hydroxide (1%, Braun, Queluz de Baixo, Barcarena, Portugal) with system water, as described in [24]. We note that propofol/lidocaine mixture is a lipid containing emulsion which is not freely soluble in water. Whilst we did not observe visible precipitation when preparing the diluted anaesthetics, we cannot rule out some reduction of the drug concentration in this way.

### 2.4 Experiment A

This experiment aimed to determine the concentration of MS-222 required to successfully anaesthetise and recover juvenile fish, monitoring their subsequent recovery. Here, we anaesthetised batches of 10 to 30 juvenile fish (sized between 7-20mm SL), according to the dish capacity, in a solution of MS-222 at several different concentrations (between 0.008% - 0.02%) for 30 minutes and then recovered them in system water. We chose 30 minutes to reflect the time required for precise orientation and mounting, plus image acquisition when using an epifluorescent or confocal microscope. Animals were randomly allocated from the offspring of more than 10 sets of zebrafish parents.

**Fig 1:**
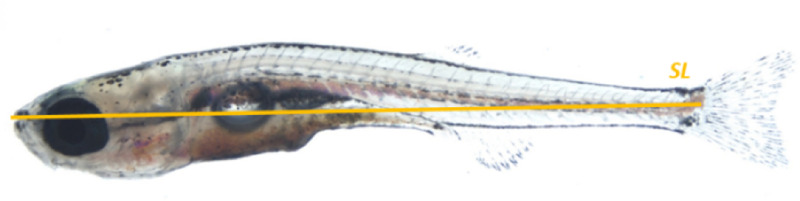
Standard length (SL) as described by Parichy *et al* [27]. SL is the distance from the snout to the caudal peduncle. In pre-flexion larvae that do not have a caudal penduncle, SL is the distance from the snout to the posterior tip of the notochord.

MS-222 was prepared as described in Section 2.2.1 and placed in a 90 mm petri dish for use. Multiple batches of fish were used to test each concentration. For each, between 10 and 30 zebrafish were collected using a plastic tea-strainer, excess water removed by briefly blotting on paper towel, and then fish transferred to the petri dish with the anaesthetic. After all the fish were transferred, they were monitored over the next 30 minutes. Fish were considered unconscious once they no longer moved freely and were not responsive to being touched gently with a mounted needle. Movement under anaesthetic (MUA) was evaluated three times; at 10, 20 and 30 minutes post immersion. Any fish was counted as MUA if a) the fish did not become anaesthetised within the first 20 minutes of being immersed in MS-222; b) the fish responded to soft touch after 10 minutes of being deemed unconscious (tested by gently touching the lateral side of the fish with forceps); or c) the fish was observed spontaneously moving after being deemed unconscious. Where fish became unconscious, they were moved by pipette to a small volume of anaesthetic (of the same dose) where the standard length (shown in Fig 1) was measured using a millimetre ruler, before returning to the petri dish for the rest of the treatment. At the end of the 30 minutes fish were transferred by plastic pipette for recovery in system water. Individuals that were slow to recover spontaneous movement were irrigated with water to aid recovery. Any fish that failed to recover within 30 minutes were noted, their length measured and then euthanized by Schedule 1 killing.

Results of fish recovery at certain concentrations informed subsequent testing. For example, if the recovery rate at a particular concentration was too low for fish of a certain length, a higher concentration was never tested on another batch of similar sized fish. Similarly if the MUA was too high at a particular concentration for fish of a certain length, a lower concentration was never tested on another batch of similar sized fish. All fish were anaesthetised and recovered in water at similar temperatures (around 28 degrees Celsius).

### 2.5 Experiment B

This experiment aimed to obtain a workable period of anaesthesia with successful recovery for fish in the sensitive juvenile stages, testing a diverse set of anaesthetics derived from the literature. We distinguished induction–the method used to initially anaesthetise the fish–from maintenance–the method used to maintain the fish under anaesthesia. We anaesthetised 60 fish individually according to one of six protocols (10 fish each) outlined in Table 1. We chose the sample size of N = 10 in accordance with other similar studies [23,24] and as a minimal but effective choice. Since we were aiming for 90–100% recovery rate, by using N = 10 fish, if the population is 90% successful, we can be 95% certain that the recovery rate lies between 70–100%. If the population was 99% successful, we could be 95% certain that the recovery rate lies between 94–100%. Informed by the results of experiment A, we reduced the time under anaesthetic to 10 minutes and diversify our anaesthetic type and method. We chose 10 minutes to reflect the time that might sometimes be required for precise orientation and mounting, plus image acquisition, e.g. using an epifluorescent microscope. Induction in all protocols and maintenance in protocols 1, 3 and 5 was the same as in experiment A: the fish were immersed in a water bath containing the anaesthetic. Fish in protocols 2, 4 and 6 were maintained under anaesthetic by being placed on a cotton pad doused in the anaesthetic. The objective of testing different maintenance methods was to see if a particular method could reduce any negative effects of using a particular anaesthetic *e*.*g*. long recovery times or spontaneous movement. Animals were randomly allocated to experimental groups from the offspring of 3 sets of adult zebrafish parents.

**Table 1:** Protocol numbers and the corresponding procedures.

Protocol	Anaesthetic	Dose	Induction	Maintenance	Time maintained (minutes)
**1**	MS-222	0.01%	Immersion	Immersion	10
**2**	MS-222	0.01%	Immersion	Cotton pad	10
**3**	2-phenoxyethanol	0.085%	Immersion	Immersion	10
**4**	2-phenoxyethanol	0.085%	Immersion	Cotton pad	10
**5**	Propofol/Lidocaine	0.00025%/0.0050%	Immersion	Immersion	10
**6**	Propofol/Lidocaine	0.00025%/0.0050%	Immersion	Cotton pad	10

At the start of each experiment, the anaesthetic of the correct concentration was prepared (as described in Section 2.2.1) and solutions were poured into a petri dish for use. If the fish was to be maintained by cotton pad, a cotton pad doused in the same anaesthetic would be placed on a second petri dish.

For each protocol, anaesthesia was induced by immersing each fish in the appropriate anaesthetic (as in Section 2.3). Fish that were to be maintained on a cotton pad (protocols 2,4,6) were removed from the anaesthetic using a plastic pipette once they had lost touch response and placed gently onto the cotton pad. At 10 minutes, the fish were removed from the anaesthetic and recovered in a petri dish of water; where fish were slow to recover spontaneous movements, water was flushed over the gills using a plastic pipette to aid recovery. Fish that did not recover within 30 minutes were recorded and their length measured, before being euthanized by Schedule 1 killing. The mean size of fish in this experiment was 9.99 mm with a standard deviation of 1.4 mm.

In contrast to experiment A, only one fish was anaesthetised in a petri dish at any given time. This allowed us to accurately measure the respiratory rate as well as the time taken for induction of anaesthesia, loss of touch response, recovery of movement and recovery of equilibrium for each individual fish.

Each fish was considered to be anaesthetised once spontaneous movements stopped, and to have lost touch response when it no longer responded to being tapped gently on the fins by a pair of blunt forceps. The respiratory rate was taken once the fish had been under anaesthetic for at least 5 minutes. The rate was measured by counting the number of movements of the mouth made in 60 seconds (*i*.*e*. breaths per minute (bpm)) under a stereomicroscope. The heart rate was taken subsequently to the respiratory rate and was measured by counting the number of times the heart pumped blood in 60 seconds under a stereomicroscope. The standard length (shown in Fig 1) was measured using a ruler. The time taken to regain movement (equilibrium) was measured as the time between the fish being placed back into fresh water and moving for the first time (regaining equilibrium and swimming freely).

### 2.6 Experiment C

Building on the results from experiment B, we here aim to identify an optimal protocol for repeated anaesthesia with successful recovery, focusing on protocols 1, 4, 5 and 6. Due to the significantly low recovery rate of protocol 3 in experiment B, we removed protocol 3 from experiment C; furthermore, an initial trial using protocol 2 revealed a high number of fish exhibiting MUA, thus protocol 2 was also removed. A preliminary trial also suggested that the dose of propofol/lidocaine used in Experiment B was too low for successful repeated anaesthesia, and thus the lidocaine dosage was doubled, informed by a recent study [24]. Thus the protocols used in Experiment C along with their corresponding dose, induction and maintenance techniques are given in Table 2. For each protocol a batch of 10 fish were anaesthetised every 4 days for 20 days for a total of 6 repeats. Again, we chose the sample size of N = 10 in accordance with other similar studies [23,24] and as a minimal but effective choice, as above. Treated fish were monitored for potential accumulated effects of anaesthetic over this period, focusing on both growth as well as changed sensitivity to the anaesthetic. Animals were randomly allocated to experimental groups from the offspring of 3 sets of adult zebrafish parents.

**Table 2:** Protocol numbers and the corresponding procedures.

Protocol	Anaesthetic	Dose	Induction	Maintenance	Time maintained (minutes)
**1**	MS-222	0.01%	Immersion	Immersion	10
**4**	2-phenoxyethanol	0.085%	Immersion	Cotton pad	10
**5**	Propofol/Lidocaine	0.00025%/0.01%	Immersion	Immersion	10
**6**	Propofol/Lidocaine	0.00025%/0.01%	Immersion	Cotton pad	10

At the start of the first repeat, all fish in all protocols were aged 21 dpf and the average size of the fish was 5.45 mm SL, 5.4 mm SL, 5.75 mm SL and 5.65 mm SL for protocols 1, 4, 5 and 6 respectively. We anaesthetised the fish in each batch as described in Experiment B. During each repeat we take the same measurements as those outlined in Experiment B. The fish was also checked for any obvious malformations or developmental retardation resulting from the repeated anaeasthesia when the breathing rate was measured. In all cases, there was no obvious malformations or developmental retardation nor any histological changes. After recovery for each repeat (except for the last) the fish were returned to the tank with the rest of the fish from the same experiment group. They remained in the fish facility with free-flowing water and feed until the next repeat. We repeated the test every four days until we had completed 6 repeats and the fish were 41 dpf. We note that in all cases, fish appeared to be behaving normally between repeated exposures i.e. fish did not move especially quickly or slowly immediately after being returned to the tank, nor 2 days later prior to being anaesthetised again. All fish that survived the six repeats were subsequently euthanized by Schedule 1 killing. One concern was whether or not fish may react to the cumulative effect of the dose *i*.*e*. the effect of repeated dosing. A confounding variable for determining whether there were any effects caused by repeated dosing was size; size would increase as the repeat number increased due to growth, and, as we had already found in experiment B, had an effect on susceptibility to the anaesthetic under some conditions. Therefore, in order to control for the effects of SL, at each time point we also performed the same experiment on 5 fish of different lengths that had not been anaesthetised before. Therefore in total we had N = 30 fish per protocol ranging in size between 8-16mm SL that were anaesthetised once, and therefore could be used to distinguish effects of repeated dose from effects of size, which we do through using multiple regression analysis.

### 2.7 Experiment D

Building on the results from experiments B and C, we test whether protocol 4, the most successful protocol of experiment C, can be used to repeatedly anaesthetise fish at more frequent intervals. In particular, we test whether juvenile (8mm SL– 16 mm SL) fish are affected by repeated anaesthesia by protocol 4 every 2 days starting at 23 dpf and for a period of 18 days (9 repeats). Animals were randomly allocated to experimental groups from the offspring of 3 sets of adult zebrafish parents. We anaesthetise the fish in each batch as described in Experiment B and C. During each repeat we take the same measurements (except for heart rate per minute and breaths per minute) as those outlined in Experiment B and C. After recovery for each repeat (except for the last) the fish were returned to individual tanks. They remained in the fish facility with free-flowing water and feed until the next repeat. We repeated the test every two days until we had completed all 9 repeats and the fish were 39 dpf. All fish that survived the nine repeats were subsequently euthanized by Schedule 1 killing. Unlike in experiment C, we did not provide a control group for the effects of SL. Since we had already determined that there were minimal effects of repeat number and SL under protocol 4, we deemed it unnecessary to control for size again in this study, thus reducing the number of animals required. As in Experiment C, for all cases, fish appeared to be behaving normally between repeated anaesthetic treatments.

### 2.8 Statistics

#### Experiment A

**Fig 2:**
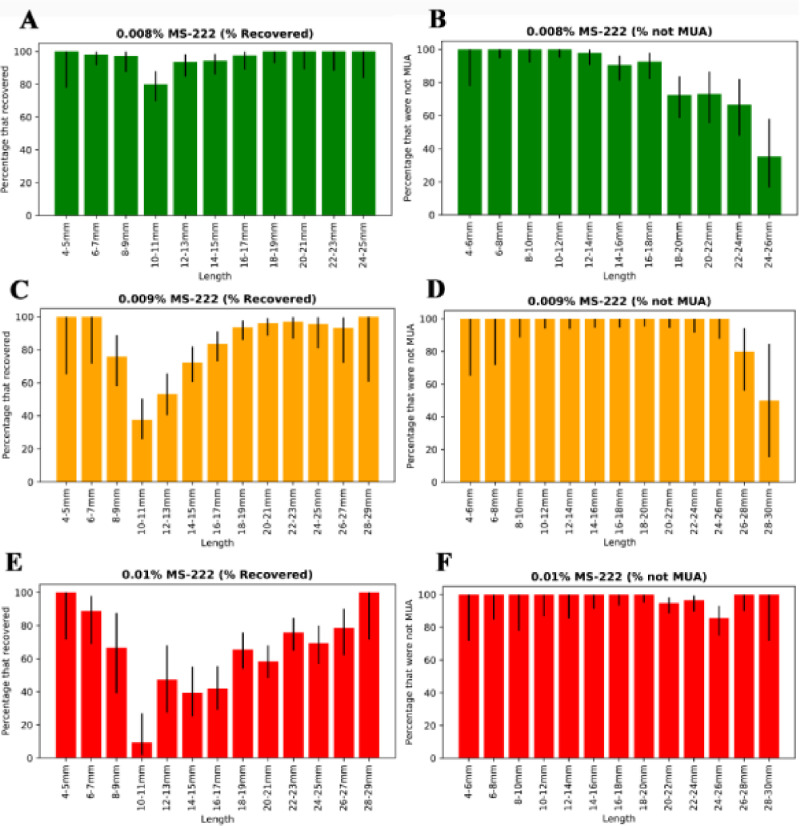
Metamorphic fish become sensitive to MS-222 around 10mm SL and later become de-sensitised around 24mm SL. (A), (C), (E) Percentage of fish that recovered by length for concentrations of 0.008%, 0.009% and 0.01% MS-222 respectively. (B), (D), (F) Percentage of fish that were not MUA for concentrations of 0.008%, 0.009% and 0.01% MS-222 respectively. Black lines represent the 90% confidence interval for each value, calculated using the sample size (see materials and methods).

To determine the 90% confidence interval for Fig 2, we used *confint*.*xls* by John C. Pezzullo (jcp12345@gmail.com), which calculates the upper and lower bound of the limit using the Clopper and Pearson method [28] before uploading as a.csv for analysis in python.

The raw data and computed confidence intervals, as well as the code for generating the bargraphs are given at: https://github.com/JenniferOwen/Experiment_A_Data_Analysis_Anaesthetic.

#### Experiments B-D

A normality test was performed for the data in experiments B, C and D, revealing that not all datasets were normally distributed. Therefore the significance test used for all bar plots was the Mann-Whitney-U test. A multi-linear regression was determined using the statistics tool in python for experiments C and D.

The code and raw data for each experiment is given as follows:

**Experiment B:** https://github.com/JenniferOwen/Experiment_B_Data_Analysis_Anaesthetic

**Experiment C:** https://github.com/JenniferOwen/Experiment_C_Data_Analysis_Anaesthetic

**Experiment D:** https://github.com/JenniferOwen/Experiment_D_Data_Analysis_Anaesthetic

## 3 Results

### 3.1 Experiment A–longer duration anaesthesia with recovery cannot be readily obtained using MS-222 for juveniles within a sensitive growth range

This experiment aimed to determine the concentration of MS-222 required to anaesthetise successfully juvenile fish, whilst ensuring their subsequent recovery. Our secondary aim was to test what impact, if any, the size of the fish (a proxy for development) has on their sensitivity to MS-222 during this period. We anaesthetised batches of 10 to 30 juvenile fish in solutions of MS-222 at several different concentrations (between 0.008% - 0.02%) for 30 minutes and then recovered them in system water. To assess possible incomplete anaesthesia, we defined Movement Under Anaesthesia (MUA) as any voluntary movement of fish made after 5 minutes of being treated with anaesthetic. If any fish exhibited MUA whilst anaesthetised under any given concentration, then this concentration was deemed too low. Similarly a concentration was considered too high if the survival rate for fish anaesthetised using that concentration was too low (<90%). Fish ‘survived’ a concentration of MS-222 if after being removed from the petri dish and recovered into fish water, they regained full movement within one hour. Since fish grow significantly bigger during this period (from 8mm-16mm SL), we also measured the standard length ([27]; Fig 1) of all fish under anaesthesia and specifically recorded the length of any fish that did not recover.

#### 3.1.1 Length, rather than age, is a better predictor of successful anaesthesia by MS-222

As has been seen previously [27], the length of larvae in each batch showed significant variation, despite all fish being of the same age and raised in the same tank. Moreover, we consistently observed that the effectiveness (ability to successfully anaesthetise and recover) of the MS-222 as an anaesthetic was dependent upon the length of the fish and not necessarily the age (despite these variables being correlated). For example, we would often observe that all fish of a certain length would not survive despite all fish being the same age. For this reason, we focused on testing a wide range of different sized fish, instead of a wide range of ages. Recovery percentage against age is shown in S1 Fig.

#### 3.1.2 Pre-metamorphic (<6mm SL) and J+ (>16mm SL) fish can be anaesthetised and recovered successfully with MS-222

We found that for fish sized between 4 mm SL and 6 mm SL, MS-222 treatment was very effective for a wide range of concentrations. For example, all fish could be anaesthetised effectively (no MUA) with any concentration from 0.008%-0.01% and >90% recovered successfully (shown in in Fig 2A–2E). In fact, pilot studies indicated that fish sized between 4mm - 6mm SL could be anaesthetised and recovered successfully with any concentration up to 0.016%. Anaesthesia was also successfully induced in fish longer than 16 mm SL. For fish sized between 16 mm SL and 26 mm SL, 0.009% MS-222 became the most suitable for effective anaesthesia and good recovery (shown in in Fig 2C and 2D).). From 26mm SL, a slightly higher dose (0.01% MS-222) became the most suitable for effective anaesthesia and simultaneous good recovery (shown in in Fig 2E and 2F).

#### 3.1.3 Metamorphic (8 mm SL– 16 mm SL) fish react unpredictably to MS-222

In contrast, fish of intermediate lengths, between 8–16 mm SL, were less predictable in their response to MS-222 treatment. For example, when using MS-222 with a concentration of 0.008%, more than 10% of fish did not recover, suggesting the dose was too high (see Fig 2A). However, when the dose was lowered to 0.007% MS-222, all fish would remain mobile within the anaesthetic, suggesting the dose was too low. Intriguingly, a small proportion of fish sized between 8 mm SL and 10 mm SL anaesthetised using 0.007% MS-222 would remain mobile throughout the procedure for 20 minutes and then subsequently die in the final 10 minutes, rendering them both MUA and non-recovery. This unpredictability continued to later stages. For example, of fish anaesthetised with 0.008% MS-222 sized between 14 mm SL—16 mm SL, more than 10% were MUA and more than 10% were non-recovery (see Fig 2A and 2B), though in this case, these were not the same fish. In general, we were unable to find a concentration of MS-222 that permitted an acceptable frequency of both successful anaesthesia (MUA) and recovery after a 30 min exposure to the anaesthetic.

### 3.2 Experiment B–alternative anaesthetics and maintenance methods have potential for repeated anaesthesia for juvenile fish

**Fig 3:**
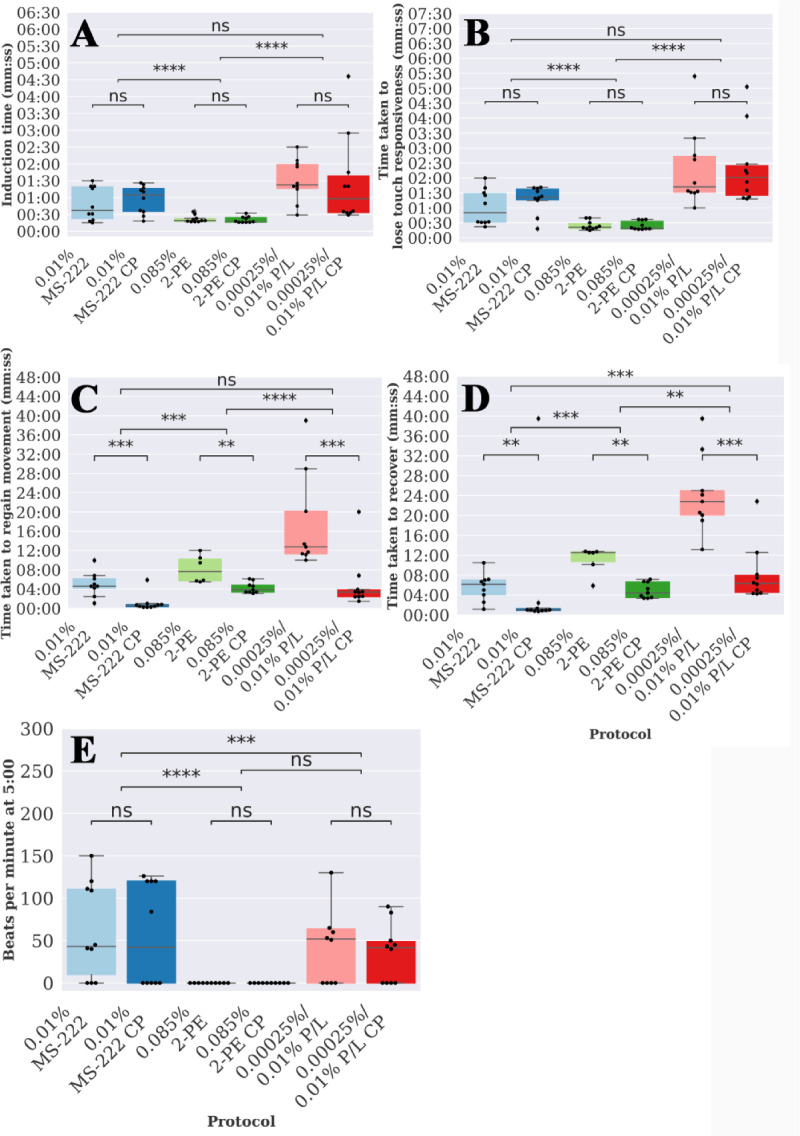
Protocols 1, 4, 5 and 6 vary in the time taken to induce, lose touch responsiveness, regain movement, regain equilibrium as well as respiratory rate. Boxplots of the time taken to (A) induce anaesthesia, (B) lose touch responsiveness, (C) regain movement post anaesthesia and (D) regain equalibrium post anaesthesia for each protocol 1–6 as defined in Table 1. (E) Boxplot of the respiratory rate (breaths per minute—bpm) at 5 minutes for each protocol. Stars indicate significant difference of subsequent repeats as determined using the Mann-Whitney U-test. ns = not significant, ‘*’ indicates p<0.05, ‘**’ indicates p<0.01, ‘***’ indicates p < 0.001 and ‘****’ indicates p<0.0001.

Consequently, we performed a pilot study of alternative anaesthetics and maintenance techniques, using a diverse set of protocols derived from the literature, with the aim to identify a potential candidate protocol for use in repeated anaesthesia of juvenile fish. In particular, we distinguished induction–the method used to initially anaesthetise the fish–from maintenance–the method used to maintain the fish under anaesthesia. A full set of protocols are described in Table 1, but consist of three different anaesthetics, with two different maintenance methods. Fish were anaesthetised for a reduced time of 10 minutes (consistent with a minimal time to image an individual fish) and then recovered in system water. To allow detailed comparison of the protocols, fish were anaesthetised individually, allowing us to monitor a series of key parameters: time taken to induce anaesthesia (i.e. stop movement), time taken to lose touch responsivity, breathing rate, time taken to regain movement post anaesthetic and time taken to fully recover (Fig 3). In all cases, there was no MUA in this experiment.

#### 3.2.1 Anaesthetic choice affects times taken to induce, lose touch response and recover post anaesthetic

Measurements of time taken for induction of anaesthesia and for loss of touch responsiveness are taken prior to the maintenance stage and are thus identical for protocols 1 and 2, 3 and 4 and 5 and 6 respectively. As expected, therefore, there is no statistically significant difference between these protocols as expected (Fig 3A and 3B). In contrast, the anaesthetic used makes a significant difference. The time taken for loss of touch response was significantly shorter (p<0.05) in the case of 2-phenoxyethanol (mean ± sd; immersion: 24 s ± 9 s), than in the case of MS-222 (1m 08 s ± 31 s), and propofol/lidocaine combination (2 m 18 s ± 1 m 17 s) (Fig 3A and 3B). Furthermore, regardless of the maintenance method, fish anaesthetised using MS-222 regained movement the quickest (immersion: 4m 58 s ±3 m 33, cottonpad: 1m 04 s ± 1 m 42), followed by 2-phenoxyethanol (immersion: 8m 09 s ± 2 m 50, cottonpad: 4m 16 s ± 1 m 09) and then by the propofol/lidocaine combination (immersion: 17 m 35 s ± 10 m 03 s, cottonpad: 4 m 58 s ± 5 m 28; Fig 3C and 3D).

#### 3.2.2 Maintenance of anaesthesia via cotton pad, as opposed to immersion, improves recovery from anaesthesia

The time taken for recovery from anaesthesia (i.e. to regain movement post anaesthesia and to regain equilibrium) were strongly affected by the maintenance method. Thus, times taken were significantly shorter (p<0.01) when the fish was maintained on a cotton pad doused in anaesthetic versus that when it remained immersed in the anaesthetic for the full 10 minutes (Fig 3C and 3D). We found no significant difference between the respiratory rates of fish maintained on a cotton pad doused in anaesthetic versus that when it remained immersed in the anaesthetic (Fig 3E). The mean respiratory rates for each protocol were similar, except that breathing ceased when anaesthetised with protocols 3 or 4. The number of fish that recovered in this pilot study was similar for the two maintenance methods with respect to MS-222 (Table 3: 9/10 and 10/10 recovery) and propofol/lidocaine (Table 3: 9/10 and 10/10). However, for 2-phenyoxyethanol the number of fish that recovered almost doubled when using a cotton pad for maintenance (Table 3: 9/10) over immersion (Table 3: 5/10).

**Table 3:** Protocol numbers and the corresponding procedures with survival percentage.

Protocol	Anaesthetic	Dose	Induction	Maintenance	Survival (%)
**1**	MS-222	0.01%	Immersion	Immersion	90
**2**	MS-222	0.01%	Immersion	Cotton pad	100
**3**	2-phenoxyethanol	0.085%	Immersion	Immersion	50
**4**	2-phenoxyethanol	0.085%	Immersion	Cotton pad	90
**5**	Propofol/Lidocaine	0.00025%/0.0050%	Immersion	Immersion	90
**6**	Propofol/Lidocaine	0.00025%/0.0050%	Immersion	Cotton pad	100

Based on the recovery rates, protocols 1,2,4,5 and 6, with 90+% recovery rates, are all suitable protocols for testing repeated anaesthesia. However, for the next stages of testing, we eliminate protocol 2 for repeated anaesthesia for juvenile stages. This is based on observations that cotton-pad maintenance appears to reduce anaesthetic strength seen in experiment B, combined with observations from experiment A that fish become less sensitive to MS-222 during later stages. We also increase the concentrations of lidocaine in protocols 5 and 6 for our repeated anaesthesia trials in order to reduce the time taken to induce anaesthesia.

### 3.3 Experiment C–towards a reliable methodology for repeated anaesthesia for juvenile fish

In this experiment we took the subset of protocols determined successful from experiment B, and tested the reliability and impacts of using these protocols for repeated anaesthesia. The full set of protocols are described in Table 2, but consist of the same protocols 1 and 4 from experiment B as well as protocols 5 and 6 with a higher concentration of lidocaine. Our aim here was to identify the best candidate protocol for use in repeated anaesthesia and imaging of juvenile fish. The criteria for this was that the protocol had to give consistent low induction and recovery times as well as minimal breathing rate to facilitate imaging. In addition, we assessed whether there were harmful cumulative effects of the anaesthetic trials, such as retarded growth, or inconvenient ones, such as prolonged time taken to induce or recover from, anaesthesia.

Ten fish for each protocol were anaesthetised for 10 minutes and then recovered in system water every 4 days from 21 dpf for a total of 20 days. As in experiment B, fish were anaesthetised individually, allowing us insight into time taken to induce anaesthesia (i.e. stop movement), time taken to lose touch responsivity, heart rate, time taken to regain movement post anaesthetic and time taken to fully recover. We also measured the length of all fish. Since we are interested in imaging the fish, we also measured the breathing rate (visible mouth movements per minute) whilst under anaesthetic, since minimal visible mouth movements would simplify the imaging requirements. We also tested to see if there were any negative effects of repeated treatment such as retarding growth, by comparing the sizes of the fish at each time point with a set of 10 control fish kept in similar conditions. We controlled for whether repeated anaesthesia had effects on individual measurements by simultaneously anaesthetising and measuring a set of fish within the same range of size and age once. Detailed documentation of our full set of results are given in the S1–S9 Figs. An ideal anaesthetic would not be significantly affected by either size or repeat number, but in this section we explore the possibility of cumulative effects of being repeatedly anaesthetised, such as adapting to the dose, or being able to flush it out more readily, or alternatively a cumulative effect leading to overdose, allowing for some effect as long as this was not detrimental. To perform this analysis, for each measurement we generated a multiple linear regression model given in the S1–S4 Tables with variables standard length and repeat number. In Table 3 we summarise, for each protocol, the effects of these variables including whether they significantly affect the outcome and if so whether this effect was positive or negative.

#### 3.3.1 Full recovery was observed for protocols 1 and 4 only

The overall recovery rate, that is, the percentage of fish that survived to the end of the full six repeats was 100% for protocols 1 and 4, 80% for protocol 6 and 60% for protocol 5 (see Table 4). The fish that did not recover in protocols 5 were all sized within the vulnerable range identified in Experiment A. In contrast, the fish that did not recover following protocol 6 were both very small. They had a mean±s.d. of 6.25±0.75 mm SL.

**Table 4:** Protocol numbers and the corresponding procedures.

Protocol	Anaesthetic	Dose	Induction	Maintenance	Survival (%)	Death occurred on repeat number:
**1**	MS-222	0.01%	Immersion	Immersion	100	N/A
**4**	2-phenoxyethanol	0.085%	Immersion	Cotton pad	100	N/A
**5**	Propofol/Lidocaine	0.00025%/0.01%	Immersion	Immersion	60	2 (1 fish)
						3 (2 fish)
						5 (1 fish)
**6**	Propofol/Lidocaine	0.00025%/0.01%	Immersion	Cotton pad	80	2 (2 fish)

#### 3.3.2 Repeated anaesthesia did not impact on fish growth

**Fig 4:**
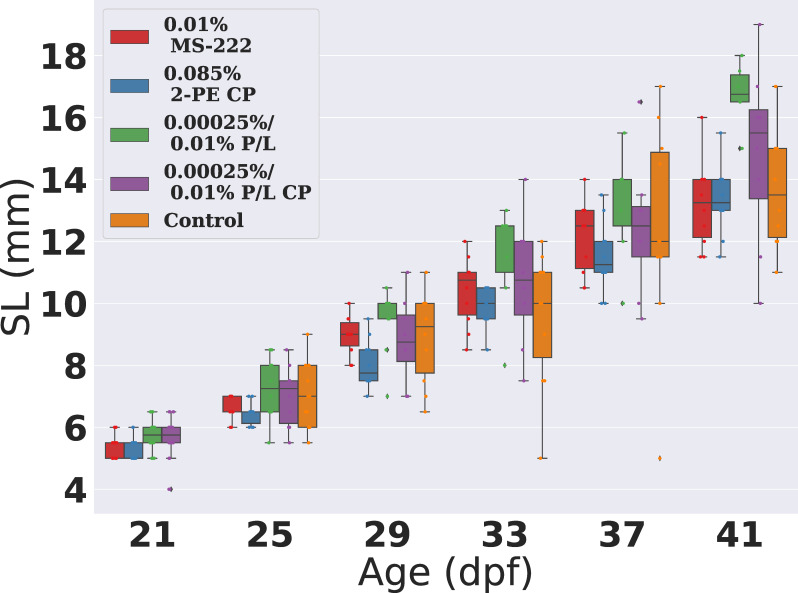
Growth of the fish is not significantly impacted by repeated dosing using any of the 4 protocols. Barchart with 95% confidence intervals for age vs length (SL (mm)) of fish anaesthetised using protocols 1,4,5 and 6 respectively.

In order to compare the development of the fish over the course of the repeated anaesthesia, we measured the lengths of fish from a control group of 10 fish at each time point. We found that in all protocols for all repeats, the size of the fish in the groups were never significantly smaller than the control groups (p>0.05), fish were either statistically similar or were significantly larger than the control, suggesting that growth of the fish is not retarded by the repeated anaesthesia (see Fig 4). Unexpectedly, at the end of the experiment fish from protocol 5 were significantly longer than the controls during later repeat numbers (p<0.05). We attribute this to the effect of attrition of fish from this group for example, from repeat number 4, fish numbers were reduced from 10 to 6. Fish kept in smaller numbers typically consume more and grow more quickly, due to decreased competition for food [29].

#### 3.3.3 Protocols 4 and 6 achieve anaesthesia most rapidly

**Fig 5:**
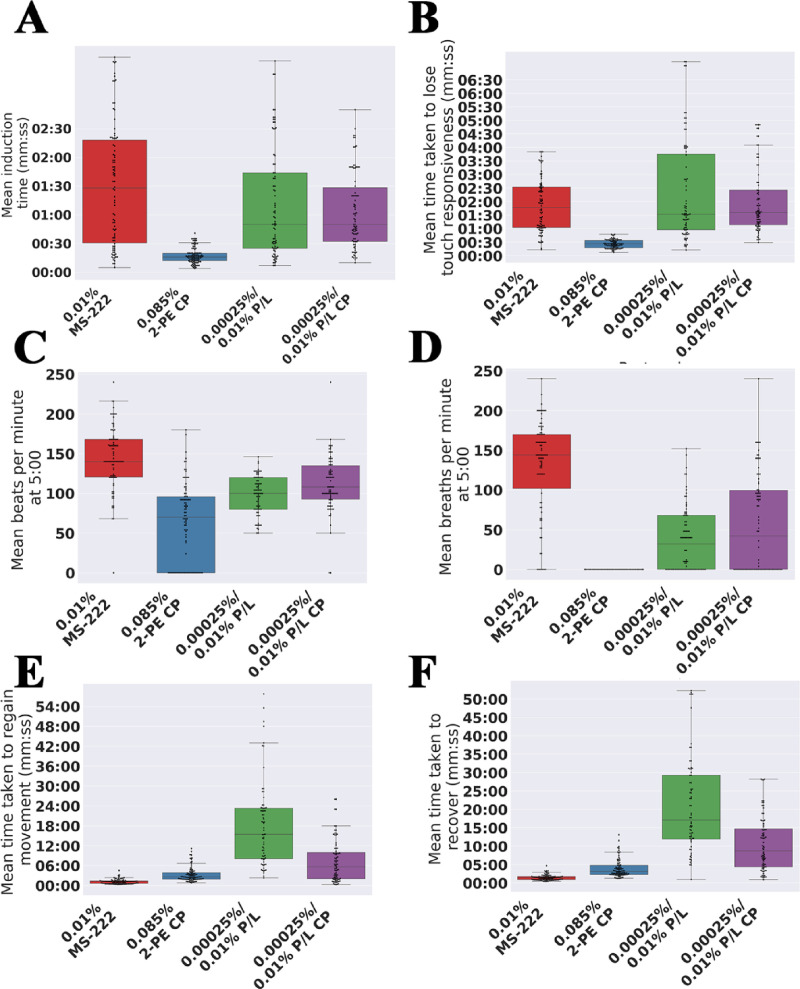
Protocols 1, 4, 5 and 6 vary in the average time taken to induce, lose touch responsiveness, regain movement, regain equilibrium as well as respiratory rate when repeatedly anaesthetised every 4 days. Barplot of the mean (A) induction time, (B) time taken to lose touch responsivity, (C) heartbeats per minute at 5:00, (D) visible breaths per minute at 5:00, (E) time taken to regain movement and (F) time taken to fully recover against repeat number for each protocol 1,4,5 and 6. Mean taken over all repeats. P/L corresponds to Propofol/Lidocaine, PE corresponds to phenoxyethanol, CP corresponds to Cotton Pad maintenance.

Since the time taken to become anaesthetised is included within the full anaesthesia time, a good anaesthetic should take a short time (<1 min) to induce anaesthesia and lose touch responsivity. Furthermore, it should not be significantly affected by repeat number and SL, or if it is significantly affected, it should be with only a small magnitude. In Fig 5A and 5B, we compare the time taken to induce anaesthesia and time taken to lose touch responsivity for all four protocols, averaged over the 6 repeats. We found that protocol 4 stands out as being the quickest both to induce anaesthesia and lose touch responsiveness (mean ± sd: 26 s ± 9 s), while all the others are slower for both measures and similar to each other. Protocol 5 is the slowest to lose touch responsiveness (mean ± sd: 1 m 54 s ± 1m 33) and is the least desirable based on time taken to achieve anaesthesia. In Table 5 we compare the effects of repeat number and SL on the time taken to induce and lose touch responsivity. We found that fish repeatedly anaesthetised using protocol 1 became quicker to achieve anaesthesia with both repeat number and SL indicating that these factors cause increased susceptibility to the uptake of MS-222. In contrast, fish repeatedly anaesthetised using protocol 4, became slower to achieve anaesthesia with repeat number and SL indicating that these factors cause decreased susceptibility to the uptake of 2-phenoxyethanol, although the magnitude of this effect is not so large. Protocols 5 and 6 were not significantly affected by repeat number, but the time taken to achieve anaesthesia decreased with SL indicating that fish become more susceptible to the propofol and lidocaine combination as the fish develops. Taken together, these data suggest protocol 4 as the most suitable based on the rapidity and reliability of achieving anaesthesia and recovery.

**Table 5:** Susceptibility to anaesthetic treatment under multiple protocols was affected by repeat number and/or standard length.

Protocol	Description	Time taken to induce and lose touch responsivity		Visible breaths per minute (bpm)		Time taken to regain movement and recover	
		Repeat number	SL	Repeat Number	SL	Repeat number	SL
**1**	MS-222 0.01% immersion	++	++	ns	+	--	ns
**4**	2-phenoxyethanol 0.085%, cottonpad.	-	-	ns	ns	--	++
**5**	Propofol/Lidocaine 0.00025%/0.01%, immersion	ns	--	ns	++	ns	--
**6**	Propofol/Lidocaine 0.00025%/0.01%, cottonpad	ns	-	--	++	ns	--

Results based on multiple linear regression models given in the supplementary material. Symbol ‘++’ indicates that the variable causes a significant positive effect and the effect is with a high magnitude *i*.*e*. >5 (s or bpm) per repeat number or 5 (s or bpm) per SL. Symbol + indicates a positive effect that is significant, without fulfilling the criteria for ‘++’. ‘ns’ indicates that the variable is not significant. Symbol ‘--’ indicates that the variable causes a significant negative effect and the effect is with a high magnitude *i*.*e*. >5 (s or bpm) per repeat number or 5 (s or bpm) per SL. Symbol--indicates a negative effect that is significant, without fulfilling the criteria for ‘--’.

#### 3.3.4 Protocol 4 suppresses breathing rate

Since we will use our method in order to image fish, a protocol that suppresses breathing rate would be desirable. Furthermore, the extent of this suppression should not be strongly affected by repeat number nor SL. In Fig 5D we compare the average breathing rate for different protocols. In contrast to the other three protocols, fish anaesthetised using protocol 4 did not visibly move their mouth during any observations. Fish anaesthetised using protocol 1 displayed the most rapid breathing (mean ± sd: 130 ± 59 breaths per minute). Curiously, fish anaesthetised using protocols 5 and 6 would breath very quickly for short periods of time (~10s) and then would stop for a long time. In Table 5 we compare the effects of repeat number and SL on breathing rate. Interestingly, breathing rate in fish anaesthetised using protocol 4 was not significantly affected by SL nor repeat number; indeed breathing rate was consistently 0 over the entire experimental time course. Meanwhile protocols 5 and 6 were significantly and strongly positively affected by SL, and protocol 5 was also significantly and strongly negatively affected by repeat number. Thus, protocol 4 stood out as being best-suited to our aims.

#### 3.3.5 Protocol 4 is most suitable method based on time taken to recover

A good anaesthetic should take a short time (<5 mins) to regain movement and fully recover. Furthermore, it should not be strongly affected by repeat number nor SL. In Fig 5E and 5F we compare the time taken to regain movement and fully recover for each of the different protocols. Fish anaesthetised using protocol 1 recovered the quickest (mean ± sd: 1 m 24 s ± 46s), followed by protocol 4 (mean ± sd: 4 m 04 s ± 2 m 31 s) and thus are the most suitable by this measure. In contrast fish anaesthetised under protocols 5 and 6 took a very long time to recover (between 15 m– 30 m). In Table 3 we compare the effects of repeat number and SL on the time taken to regain movement and recover. Interestingly all protocols were significantly affected by one or both of SL and or repeat number with a high magnitude. Fish anaesthetised using protocol 1 were negatively affected by repeat number but not SL, suggesting that repeated dosing may help the fish to flush MS-222 from their system. In contrast, fish anaesthetised using protocols 5 and 6 were negatively affected by repeat number but not SL, indicating that as fish develop they may become more efficient at removing the propofol/lidocaine mix from their system. Interestingly the time taken for fish anaesthetised by protocol 4 to recover was negatively affected by repeat number and positively affected by SL at a similar magnitude. Since between each repeat fish grew on average 1mm, these effects essentially cancel out. Therefore, protocol 4 is the most suitable protocol for repeated anaesthesia, based on the time taken to recover.

#### 3.3.6 Maintenance of anaesthesia via cotton pad, as opposed to immersion, improves recovery and time taken to recover from anaesthesia for propofol/lidocaine mix

Protocols 5 and 6 both use a propofol/lidocaine mix described in Table 2, except in protocol 5 we maintain fish in anaesthetic solution, whereas for protocol 6 we maintain fish on a cotton pad soaked in anaesthetic. We found that the time taken to recover is approximately halved using the cotton pad versus maintenance in anaesthetic (mean ± sd: 9 m 07 s ± 6 m 03 versus 21 m 19 s ± 15 m 16 s) suggesting that the maintenance method of a cotton pad helps to reduce the effects of the anaesthetic during the recovery period. Consistent with this, we also found that the recovery rate was higher for protocol 6 over protocol 5.

#### 3.3.7 Protocol 4 is the most suitable protocol for repeated anaesthesia

In Table 6 we compare the different protocols by the success measures given in this section. Meeting 7/8 of the criteria, we deem that protocol 4 is substantially the most suitable for repeated anaesthesia under the conditions tested. Protocol 1 is second, meeting 4/8 criteria, and would be suitable for repeated anaesthesia that did not require imaging. In contrast, we deem that protocols 5 and 6 are not suitable for repeated anaesthesia during the juvenile period tested. Fish anaesthetised using these protocols took a long time to be anaesthetised and recover, visibly breathed frequently and there was not 100% recovery.

**Table 6:** Summary table of protocols and successes.

Protocol	1	4	5	6
**Average time taken to induce and lose touch responsivity quick (<1m)**	X	✓	X	X
**Time taken to induce touch responsivity not affected by SL and repeat number with a high magnitude**	X	✓	✓	X
**Breaths per minute <10**	X	✓	X	X
**Breaths per minute not affected by SL and repeat number with a high magnitude**	✓	✓	X	X
**Time taken to recover and regain movement and recover (<5m)**	✓	✓	X	X
**Time taken to recover and regain movement not effected by SL and repeat number with a high magnitude**	X	X	X	X
**Growth not retarded by repeated dose**	✓	✓	✓	✓
**100% recovery**	✓	✓	X	X
**Total score**	4/8	7/8	2/8	1/8

Protocol 4 has by far the highest score of all protocols.

### 3.4 Experiment D–protocol 4 is effective for frequent, repeated anaesthesia with recovery throughout the susceptible juvenile period

Finally, we performed a focussed study to assess the impact of frequent, repeated anaesthesia with recovery using protocol 4. A batch of 10 fish were repeatedly anaesthetised every 2 days, starting from 23 dpf for a total of 9 repeats (18 days). Unlike in experiment C wherein all 10 fish were housed in one tank throughout the experiment due to space constraints, we housed the fish individually, allowing us to track individual development and anaesthesia effect on fish individually. We note that there was no significant difference between the measured effects of individual fish.

As in experiment C, we recorded the time taken to induce anaesthesia (i.e. stop movement), time taken to lose touch responsivity, time taken to regain movement post anaesthetic and time taken to fully recover, as well as the length of the fish. We did not measure heart or breathing rate. We tested whether fish experience retarded growth over the repeats by comparing the sizes of the fish at the start and end point with a set of 10 control fish also housed individually. As we had already established that there were no adverse effects of repeated anaesthetic using protocol 4 in experiment C, in order to minimise the number of fish needing to be involved in this trial we did not control for the effects of repeated versus single anaesthesia. The aim of this experiment was to identify whether protocol 4 was suitable for repeated anaesthesia with more frequent dosing, *i*.*e*. that the fish could be anaesthetised and recovered quickly, did not breathe frequently, that there was no MUA and all fish would be recovered, whilst also avoiding developmental retardation.

#### 3.4.1 Growth of the fish was not retarded by the repeated anaesthesia at a higher frequency

In order to compare the development of the fish over the course of the repeated anaesthesia, at the start of the experiment we separated 20 fish from an initial batch of 50 fish with the same age and similar size that had been kept in a tank together since 5 dpf. Of these 20 fish, 10 fish were chosen (uniformly at random) to occupy a control group and the rest were allocated to the experimental group. All fish were housed in individual tanks during the 20 day period. Fish in the experimental group were measured during every anaesthetic trial. Fish in the control group were measured once, at the experimental end point, after 18 days. The mean±s.d.length of the experimental and control fish at 31 dpf showed no significant difference (experimental, 16.35±2.11 mm SL; control, 17.45±1.9 mm SL.

**Fig 6:**
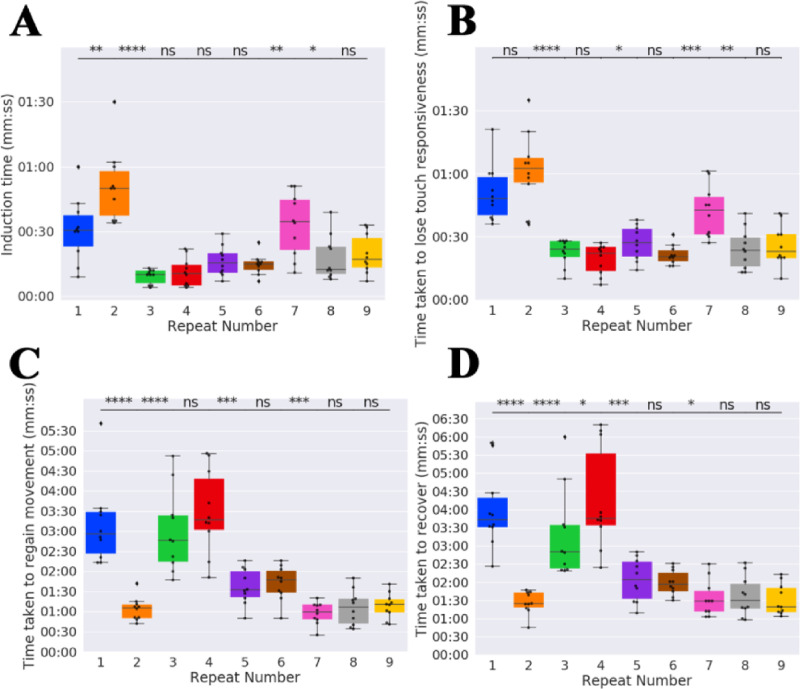
The time taken to induce, lose touch responsiveness, regain movement, regain equilibrium as well as respiratory rate varies within an acceptable range for fish repeatedly anaesthetised using protocol 4 every 2 days. Boxplot of (A) induction time, (B) time taken to lose touch responsiveness, (C) time taken to regain movement and (D) time taken to recover. Stars indicate significant difference of subsequent repeats as determined using the Mann-Whitney U test. ns = not significant, ‘*’ indicates p<0.05, ‘**’ indicates p<0.01, ‘***’ indicates p < 0.001 and ‘****’ indicates p<0.0001.

*Protocol 4 remained an efficient and effective method for inducing anaesthesia with recovery when used frequently*. We found that increased frequency of inducing anaesthesia showed no significant impact on the suitability of protocol 4 Just as when fish were repeatedly anaesthetised every 4 days, the time taken to induce anaesthesia (~1 min), and to recover from anaesthesia remained low (~5 min see Fig 6A and 6B). Breathing rate was consistently very low. Finally, we compare the effects of SL and repeat number on the time taken to induce and the time taken to recover for fish repeatedly anaesthetised using protocol 4 (Table 7). Interestingly, when the fish were repeatedly anaesthetised every 2 days, SL had a positive effect rather than a negative effect on the time taken to induce anaesthesia. Furthermore, unlike when fish were anaesthetised every 4 days, the time taken to regain movement and recover was not significantly affected by either repeat number of SL.

**Table 7:** Comparison of the significance of repeat number and standard length on the time taken to induce and lose touch responsivity, breathing rate and time taken to regain movement and recover for protocol 4 when used every 2 days vs every 4 days.

	Time taken to induce and lose touch responsivity		Breaths per minute		Time taken to regain movement and recover	
**Protocol 4**	Repeat number	SL	Repeat Number	SL	Repeat number	SL
**Every 4 days**	-	-	ns	ns	--	++
**Every 2 days**	-	+	ns	ns	ns	ns

Results based on multiple linear regression models given in the supplementary material. Symbol ‘++’ indicates that the variable causes a significant positive effect and the effect is with a high magnitude *i*.*e*. >5 (s or bpm) per repeat number or 5 (s or bpm) per SL. Symbol + indicates a positive effect that is significant, without fulfilling the criteria for ‘++’. ‘ns’ indicates that the variable is not significant. Symbol ‘--’ indicates that the variable causes a significant negative effect and the effect is with a high magnitude *i*.*e*. >5 (s or bpm) per repeat number or 5 (s or bpm) per SL. Symbol--indicates a negative effect that is significant, without fulfilling the criteria for ‘--’.

#### 3.4.2 Protocol 4 is a suitable protocol for repeated, frequent anaesthesia

Based on these new results, in Table 8 we assess protocol 4 using the same criteria as for experiment C. We observe that protocol 4 is successful in fulfilling all criteria when used for repeated anaesthetic every 2 days. Therefore we conclude that protocol 4 is a suitable protocol for frequent repeated anaesthesia during juvenile periods.

**Table 8:** Summary table of successes for protocol 4 for two different frequencies.

Protocol 4	Every 4 days	Every 2 days
**Average time taken to induce and lose touch responsivity quick (<1m)**	✓	✓
**Time taken to induce touch responsivity not affected by SL and repeat number with a high magnitude**	✓	✓
**Breaths per minute <10**	✓	✓
**Breaths per minute not affected by SL and repeat number with a high magnitude**	✓	✓
**Average time taken to recover and regain movement and recover (<5m)**	✓	✓
**Time taken to recover and regain movement not effected by SL and repeat number with a high magnitude**	X	✓
**Growth not retarded by repeated dose**	✓	✓
**100% recovery**	✓	✓
**Total score**	7/8	8/8

In both cases, protocol 4 has a high score and thus is a suitable protocol for repeated anaesthesia.

## 4 Discussion

Whilst there have been some studies of anaesthesia protocols as applied to adult and larval zebrafish [14,23,24,30], there have been none to our knowledge that have tested anaesthetics during the juvenile period.

Zebrafish larvae and adult fish vary greatly in their response to anaesthetics due to their significantly different physiologies [31,32]. Adult zebrafish rely on their gills for oxygen absorption. The usual cause of death for adult fish under anaesthesia is by asphyxiation, caused by the blockage of gill ventilation leading to hypoxemia [33,34]. Deeply anaesthetised adult fish can be kept alive for hours to days, but only if their gills are artificially ventilated [35]. Zebrafish larvae do not have gills and thus rely on cutaneous gas exchange for oxygen absorption. Zebrafish larvae, as a result, are considerably more tolerant to the lethal effects of MS-222 than adult fish [36].

Anaesthetic treatment for juvenile stages therefore holds uncertainty. During the juvenile period, metamorphosis includes substantial changes in anatomy and physiology, including of the gills. These start to develop between 5 dpf and 33 dpf [31]. Zebrafish switch from absorbing oxygen by cutaneous gas exchange [37], to gill absorption at this stage. Therefore, it is logical to expect a change in tolerance to the lethal effects of anaesthetics during this period. Therefore, our aim was to refine the method for anaesthetising juvenile fish to maximise the welfare during this poorly characterised period of development.

Initially, we studied the effect of different concentrations of the most commonly used fish anaesthetic: MS-222 on the induction of anaesthesia for 30 minutes exposure and subsequent recovery of zebrafish at juvenile stages. We found that we were unable to determine any dose for zebrafish between the ages of 21 dpf and 60 dpf, such that > 90% fish could be both anaesthetised for 30 minutes and also be successfully recovered. We found that survival of the fish for a given concentration of MS-222 varied with the length of the zebrafish, confirming earlier observations that this provided a more accurate measure of ‘developmental stage’ than age [38].

We identified a critical period related to size, between 8–16 mm SL (loosely corresponding to approx. 25–45 dpf) when zebrafish experience enhanced sensitivity to MS-222. During this period, fish would remain active in a dose of 0.007% MS-222 and yet were vulnerable to being euthanized by a dose of 0.008% MS-222. In some cases, fish would move spontaneously for up to 20 minutes in the anaesthetic, stop moving abruptly and then die a few minutes later. We have subsequently identified one other report that noted that zebrafish aged between 21–70 dpf are particularly sensitive to MS-222 [9]. We are unsure of the precise cause of death for fish during this period. One possible reason could be linked to gill development during this period. At an approximately equivalent stage (23–33 dpf), gill filaments transform from a basic branched shape to a structure close to their final definitive form [31]. During this time, the overall contribution of cutaneous gas exchange to oxygen uptake decreases and the gills become the more dominant site for gas intake. It is possible that the changes which occur during this period leave the fish more vulnerable to the lethal effects of MS-222 by asphyxiation.

We next aimed to determine a suitable anaesthesia and recovery protocol for this period. We reduced the duration of anaesthesia required to 10 mins. Furthermore, we broadened our anaesthetic trial to include two other anaesthetics that have received attention recently by the zebrafish community as well as a medium 0.01% dose of MS-222. The combination of propofol and lidocaine was recently tested and recommended for adult zebrafish in a study by Valentim *et al*. [24]. Propofol is short-acting, rapidly metabolized and less prone to cumulative effects than MS-222 [30]. It is generally considered a very safe drug in other vertebrates and mammals and is commonly used in veterinary medicine, though not often in fish. It has a biological half-life in rainbow trout of 1.1 hours at 17°C [39] (the estimated plasma half-life of MS-222 is between 1.5 hours and 4.0 hours [40]). Propofol is combined with lidocaine to achieve analgesia. The combination also allows for a decrease in the propofol dose, resulting in a safer analgesia and anaesthesia. We also included a dose of 0.085% 2-phenoxythanol. 2-Phenoxyethanol is commonly used in aquaculture [14] and shows a rapid effect and recovery time. It has a reduced physiological effect when compared with MS-222 and has been recommended in some literature as a suitable alternative to MS-222 for anaesthesia of adult fish [18]. In adult fish, 2-phenoxyethanol is absorbed through the gills and transported via the arterial blood to the CNS. It is rapidly excreted (via the gills) and has a biological half-life in rainbow trout of less than 30 minutes. 2-phenoxyethanol is considered suitable for aquaculture because of its ease of preparation, low cost, rapid action and fast, uneventful recovery [25]. When left for extensive periods of 96–140 hours, 2-phenoxyethanol has been shown to be toxic for zebrafish embryos (< 5 dpf, 140h LC50: 461.52–521.55 mg.L^–1^) and fish sized between 30 ± 5 mm SL (96h LC50: 312.10–349.02 mg.L^–1^) [41]. 2-phenoxyethanol has not been tested for juvenile stages between 8 m SL -16 mm SL and not for the much shorter times considered here. It should be noted that health concerns have been raised about repeated exposure to this compound on the handler [42]; these would suggest that caution should be exercised and specifically that fish at other stages of development should be treated with MS-222 as is current standard practise. For each anaesthetic, we tested two different methods of anaesthesia maintenance. Our aim was to determine the best anaesthetic procedures for juvenile fish, with the plan to take those forward to the next stage of testing consisting of repeated anaesthesia. We found that for all protocols, all fish were successfully anaesthetised and once anaesthetised, remained non-touch responsive (non MUA) throughout the procedure. This suggests that all the anaesthetics trialled in experiment B were appropriate for successful anaesthesia in juvenile zebrafish.

Of the three anaesthetics, 2-phenoxyethanol had the lowest survival rate–across the two methods, 30% of the fish did not recover post-anaesthesia. The time taken to induce and lose touch responsiveness for these fish was significantly shorter than for fish anaesthetised using MS-222 and the propofol/lidocaine combination. This suggests that 2-phenoxyethanol has a stronger effect on juvenile zebrafish than the other two anaesthetics. Fish anaesthetised using the propofol/lidocaine combination were the slowest to be anaesthetised and the slowest to recover. This suggests that the combination is slow acting yet strongly perjuring for juvenile fish. Our recorded times for this protocol were similar to those observed by Valentim *et al*. [24] when tested in adult zebrafish. Fish anaesthetised using MS-222 were the quickest to recover. We found the reduction in time spent under anaesthetic–from 30 minutes to 10 minutes–greatly increased the probability of recovery. In experiment A, the proportion of fish sized between 8 mm SL and 16 mm SL that recovered from 30 minutes of 0.01% MS-222 concentration varied from 10% (10 mm SL– 12 mm SL) to 70% (8 mm– 10 mm SL). When the time spent under anaesthesia was reduced to 10 minutes, as in experiment B protocol 1, we observed a high recovery rate of 90% for fish sized between 8 mm SL and 16 mm SL. This suggests that a shorter anaesthetic time is preferential for juvenile fish.

Consistently, we found that fish maintained on a cotton pad were quicker to regain movement and recover equilibrium, compared to fish that were kept in the bath of anaesthesia. This is likely due to a smaller surface area in contact with the anaesthetic. Fish anaesthetised by 2-phenoxyethanol had an increased recovery rate from 50% to 90% when maintained on a cotton pad, suggesting that maintenance by cotton pad can increase non-lethal anaesthesia time for strong anaesthetics. However, another reason could be related to the oxygen availability. One study suggests that the pectoral fins play an important role in mixing up the oxygen boundary layer and hence maintaining high oxygen levels at the skin surface. For example, when early zebrafish larvae aged between 4–20 dpf were anaesthetised to briefly halt fin and gill ventilation, the oxygen concentration in the water near the fins decreased by as much as 50% [43]. Therefore, cotton pad maintenance may play a role in maintaining a high oxygen level at the skin surface at stages when fish are unable achieve efficient gas exchange using their gills. It is not clear whether there are any welfare implications of using one method over another. The cotton pad method could in theory be stressful for the fish as they are outside of their natural environment, however, we found no evidence that would indicate that the animals were more stressed. For example, there was no increase in the breathing or heart rate, known signs of stress in fish [44], using the cotton pad method. We note that if our prediction that the cotton pad method reduces fatality by allowing a better oxygen supply to the fish, an equivalent method would be to partially submerge the fish in the anaesthetic. This method may be more suitable for some imaging purposes, e.g. with transmitted light.

In experiment C, we aimed to determine whether cumulative exposure of remaining methods may produce any negative side effects due to accumulation of the anaesthetic.

Previous investigation of repeated use of MS-222 to anaesthetise fish is limited and does not encompass zebrafish. However, such investigations have suggested that repeated exposure to MS-222 could inhibit development in some fish. For example, when hybrid tilapias were exposed weekly to MS-222, the fish displayed significant reductions in development upon the third exposure and thereafter [45], suggesting that repeated doses of MS-222 may accumulate. Consistent with this, Atlantic salmon repeatedly anaesthetized with MS-222 exhibited upregulation of osmoregulatory genes in the gill and effects on blood parameters [46]. MS-222 anaesthetised rainbow trout have also been shown to ingest 15–20% less food than non-anaesthetised fish for up to 48 hours post anaesthesia [13]. Food plays an important role in timely development. For maximum weight gain during juvenile development, protein intake is recommended to be 14mg/g average body weight/day [47]. If MS-222 also inhibits feeding in juvenile fish, repeated exposure could greatly stunt food intake and hence arrest development. There is currently no available research into the effects of repeated exposure to propofol/lidocaine combination nor of 2-phenoxyethanol in fish. Despite these concerns, we found that for all protocols the fish grew at a rate comparable to our control group, suggesting that repeated anaesthesia once every 4 days has no impact on development. We found that MS-222 maintained via immersion is suitable for repeated anaesthesia of juvenile fish when used at a concentration of 0.01% for 10 minutes every 4 days. This protocol had a 100% survival rate, did not noticeably impact development and showed a very small effect of de-sensitisation to the anaesthetic over many repeats.

2-Phenoxyethanol maintained on a cotton pad is also suitable for repeated anaesthesia of juvenile fish when used at a concentration of 0.085% for 10 minutes every 4 days. This protocol had a 100% survival rate, did not impact development and showed a very small effect of de-sensitisation to the anaesthetic over many repeats. Repeated exposure to 2-phenoxyethanol has been shown to affect the anaesthetic’s pharmacokinetics in juvenile Angelfish, causing some tolerance; it is not clear whether other fish species may also develop tolerance to 2-phenoxyethanol. 2-phenoxyethanol has been shown to cause a stress response and immunodepression in adult seabream [48]. We found no obvious signs that the zebrafish was stressed during the procedure–no rapid breathing for example. An advantage of this method was the very low breath count, making it highly suitable for technique that require fish to stop moving completely, such as repeated imaging. Therefore, out of protocols 1 and 4, protocol 4 was the most suitable for repeated anaesthesia for imaging, due to fish exhibiting a suppression of breathing movements with this method.

In contrast, we conclude that protocols 5 and 6 were not suitable for repeated anaesthesia during this sensitive period. Firstly, under these protocols we observed a less than 100% recovery. Furthermore, fish were vibration responsive throughout the experiments, making them inappropriate for repeated anaesthesia for imaging. Also, unlike fish anesthetised under protocols 1 and 4, which either did not breath during anaesthesia (protocol 4), or did so at a controlled and constant pace (protocol 1), fish anaesthetised under protocols 5 and 6 alternated between not moving their mouth, to opening and closing their mouth rapidly. Further work is required to determine the cause of these differences. One reason could be a lack of oxygen. Opening and closing of the mouth, as well as gill movement is part of the respiratory cycle [49]. Rapid gill movements in zebrafish larvae, is associated with hypoxia [43].

Finally, in Experiment D, we showed that protocol 4 remained suitable for repeated anaesthesia with recovery, even when the frequency of anaesthesia was increased from every 4 days to every 2 days. From this experiment we deduce that protocol 4 is suitable for frequent repeated anaesthesia during the juvenile period.

## 5 Conclusions

In conclusion, we have shown that during the juvenile period (between 6 mm SL and 30 mm SL), the MS-222 dose (administered via water bath) required to successfully anaesthetise zebrafish is dependent on the length of the fish. Moreover, for some sensitive periods (8 mm SL– 16 mm SL) there is no suitable standard dose which can successfully anaesthetise for 30 minutes with both no MUA and full recovery. In contrast, zebrafish sized between 8 mm SL and 16 mm SL can be successfully anaesthetised for 10 minutes in a water bath using MS-222 at a concentration of 0.01% and maintained by immersion (protocol 1). Fish of this size can also be successfully anaesthetised using 2-phenoxyethanol at a concentration of 0.085% when maintained on an anaesthetic doused cotton pad (protocol 4). Protocols 1 and 4 are also successful methods (i.e. 100% recovery, low induction time and recovery times) when used to repeatedly anaesthetise juvenile fish every 4 days (for 6 repeats). Furthermore, repeated anaesthesia of zebrafish over this developmental period does not necessarily effect development. Of the two protocols, protocol 4 is a better protocol for imaging as fish do not visibly move their mouth during anaesthesia. Furthermore, protocol 4 is a suitable method for repeatedly anaesthetising fish when the frequency is increased to every 2 days (for 9 repeats).

Therefore, based on our data presented here, we propose that 2-phenoxyethanol at a concentration of 0.085% maintained using a cotton pad doused in anaesthetic provides the best currently available protocol for repeated anaesthesia with recovery during the 8–16 mm SL sensitive stages, offering a refinement over other adult-focussed protocols.

## Supporting information

S1 Fig Age is not a good predictor of recovery. (A), (B), (C) Percentage of fish that recovered by age for concentrations of 0.008%, 0.009% and 0.01% MS-222 respectively. Black lines represent the 90% confidence interval for each value, calculated using the sample size (see materials and methods).
(PDF)

S2 Fig The time taken to induce, lose touch responsiveness, regain movement, recover as well as respiratory rate varies with repeat number when repeatedly anaesthetised using protocol 1 every 4 days. Boxplot of (A) induction time, (B) time taken to lose touch responsivity, (C) heartbeats per minute at 5:00, (D) visible breaths per minute at 5:00, (D) time taken to regain movement and (E) time taken to fully recover against repeat number when using Protocol 1 to repeatedly anaesthetise and recover fish. Stars indicate significant difference as determined using the Mann-Whitney U test. ns = not significant, ‘*’ indicates p<0.05, ‘**’ indicates p<0.01, ‘***’ indicates p < 0.001 and ‘****’ indicates p<0.0001.
(PDF)

S3 Fig The time taken to induce, lose touch responsiveness, regain movement, recover as well as respiratory rate varies with SL when repeatedly anaesthetised using protocol 1 every 4 days. Scatter plot of (A) induction time, (B) time taken to lose touch responsivity, (C) beats per minute at 5:00, (D) breaths per minute at 5:00, (E) time taken to regain movement and (F) time taken to fully recover against repeat number over their standard length (mm). Blue dots correspond to fish that were repeatedly dosed. Red dots correspond to fish that were dosed once (*i*.*e*. control group). Blue and red linear regression lines are superimposed with translucent 95% confidence interval.
(PDF)

S4 Fig The time taken to induce, lose touch responsiveness, regain movement, recover as well as respiratory rate varies with repeat number when repeatedly anaesthetised using protocol 4 every 4 days. Boxplot of (A) induction time, (B) time taken to lose touch responsivity, (C) heartbeats per minute at 5:00, (D) visible breaths per minute at 5:00, (D) time taken to regain movement and (E) time taken to fully recover against repeat number when using Protocol 4 to repeatedly anaesthetise and recover fish. Stars indicate significant difference as determined using the Mann-Whitney U test. ns = not significant, ‘*’ indicates p<0.05, ‘**’ indicates p<0.01, ‘***’ indicates p < 0.001 and ‘****’ indicates p<0.0001. CP stands for cotton pad maintenance method.
(PDF)

S5 Fig The time taken to induce, lose touch responsiveness, regain movement, recover as well as respiratory rate varies with SL when repeatedly anaesthetised using protocol 4 every 4 days. Scatter plot of (A) induction time, (B) time taken to lose touch responsivity, (C) beats per minute at 5:00, (D) breaths per minute at 5:00, (E) time taken to regain movement and (F) time taken to fully recover against repeat number over their standard length (mm). Blue dots correspond to fish that were repeatedly dosed. Red dots correspond to fish that were dosed once (*i*.*e*. control group). Blue and red linear regression lines are superimposed with translucent 95% confidence interval.
(PDF)

S6 Fig The time taken to induce, lose touch responsiveness, regain movement, recover as well as respiratory rate varies with repeat number when repeatedly anaesthetised using protocol 5 every 4 days. Boxplot of (A) induction time, (B) time taken to lose touch responsivity, (C) heartbeats per minute at 5:00, (D) visible breaths per minute at 5:00, (D) time taken to regain movement and (E) time taken to fully recover against repeat number when using Protocol 4 to repeatedly anaesthetise and recover fish. Stars indicate significant difference as determined using the Mann-Whitney U test. ns = not significant, ‘*’ indicates p<0.05, ‘**’ indicates p<0.01, ‘***’ indicates p < 0.001 and ‘****’ indicates p<0.0001. CP stands for cotton pad maintenance method.
(PDF)

S7 Fig The time taken to induce, lose touch responsiveness, regain movement, recover as well as respiratory rate varies with SL when repeatedly anaesthetised using protocol 5 every 4 days. Scatter plot of (A) induction time, (B) time taken to lose touch responsivity, (C) beats per minute at 5:00, (D) breaths per minute at 5:00, (E) time taken to regain movement and (F) time taken to fully recover against repeat number over their standard length (mm). Blue dots correspond to fish that were repeatedly dosed. Red dots correspond to fish that were dosed once (*i*.*e*. control group). Blue and red linear regression lines are superimposed with translucent 95% confidence interval.
(PDF)

S8 Fig The time taken to induce, lose touch responsiveness, regain movement, recover as well as respiratory rate varies with repeat number when repeatedly anaesthetised using protocol 6 every 4 days. Boxplot of (A) induction time, (B) time taken to lose touch responsivity, (C) heartbeats per minute at 5:00, (D) visible breaths per minute at 5:00, (D) time taken to regain movement and (E) time taken to fully recover against repeat number when using Protocol 4 to repeatedly anaesthetise and recover fish. Stars indicate significant difference as determined using the Mann-Whitney U test. ns = not significant, ‘*’ indicates p<0.05, ‘**’ indicates p<0.01, ‘***’ indicates p < 0.001 and ‘****’ indicates p<0.0001. CP stands for cotton pad maintenance method.
(PDF)

S9 Fig The time taken to induce, lose touch responsiveness, regain movement, recover as well as respiratory rate varies with SL when repeatedly anaesthetised using protocol 6 every 4 days. Scatter plot of (A) induction time, (B) time taken to lose touch responsivity, (C) beats per minute at 5:00, (D) breaths per minute at 5:00, (E) time taken to regain movement and (F) time taken to fully recover against repeat number over their standard length (mm). Blue dots correspond to fish that were repeatedly dosed. Red dots correspond to fish that were dosed once (*i*.*e*. control group). Blue and red linear regression lines are superimposed with translucent 95% confidence interval.
(PDF)

S1 Table The effects of repeat number and SL on success measures for protocol 1 when used every 4 days. Multiple linear regression model for y = induction time, time taken to lose touch responsiveness, beats per minute, breaths per minute, time taken to regain movement and time taken to recover under protocol 1. For each y value a significant regression equation was found if p<0.05 and can be described as (F(Df model, Df residual) = F-stat. The predicted y value is given by y = C+m_1_*(repeat number)+m_2_*(standard length), where repeat number is the number of doses the fish will have been exposed to at the end of the experiment and standard length is in mm. Values highlighted are those where the associated p-value is <0.05 and thus can be deemed as significant.
(DOCX)

S2 Table The effects of repeat number and SL on success measures for protocol 4 when used every 4 days. Multiple linear regression model for y = induction time, time taken to lose touch responsiveness, beats per minute, breaths per minute, time taken to regain movement and time taken to recover under protocol 1. For each y value a significant regression equation was found if p<0.05 and can be described as (F(Df model, Df residual) = F-stat. The predicted y value is given by y = C+m_1_*(repeat number)+m_2_*(standard length), where repeat number is the number of doses the fish will have been exposed to at the end of the experiment and standard length is in mm. Values highlighted are those where the associated p-value is <0.05 and thus can be deemed as significant.
(DOCX)

S3 Table The effects of repeat number and SL on success measures for protocol 5 when used every 4 days. Multiple linear regression model for y = induction time, time taken to lose touch responsiveness, beats per minute, breaths per minute, time taken to regain movement and time taken to recover under protocol 1. For each y value a significant regression equation was found if p<0.05 and can be described as (F(Df model, Df residual) = F-stat. The predicted y value is given by y = C+m_1_*(repeat number)+m_2_*(standard length), where repeat number is the number of doses the fish will have been exposed to at the end of the experiment and standard length is in mm. Values highlighted are those where the associated p-value is <0.05 and thus can be deemed as significant.
(DOCX)

S4 Table The effects of repeat number and SL on success measures for protocol 6 when used every 4 days. Multiple linear regression model for y = induction time, time taken to lose touch responsiveness, beats per minute, breaths per minute, time taken to regain movement and time taken to recover under protocol 1. For each y value a significant regression equation was found if p<0.05 and can be described as (F(Df model, Df residual) = F-stat. The predicted y value is given by y = C+m_1_*(repeat number)+m_2_*(standard length), where repeat number is the number of doses the fish will have been exposed to at the end of the experiment and standard length is in mm. Values highlighted are those where the associated p-value is <0.05 and thus can be deemed as significant.
(DOCX)

S5 Table The effects of repeat number and SL on success measures for protocol 4 when used every 2 days. Multiple linear regression model for y = induction time, time taken to lose touch responsiveness, beats per minute, breaths per minute, time taken to regain movement and time taken to recover under protocol 4 when used every 2 days. For each y value a significant regression equation was found if p<0.05 and can be described as (F(Df model, Df residual) = F-stat. The predicted y value is given by y = C+m_1_*(repeat number)+m_2_*(standard length), where repeat number is the number of doses the fish will have been exposed to at the end of the experiment and standard length is in mm. Values highlighted are those where the associated p-value is <0.05 and thus can be deemed as significant.
(DOCX)
