# Supplementary material for: A suitable anaesthetic protocol for metamorphic zebrafish
Source: PLoS One. 2021 Mar 5;16(3):e0246504. doi: 10.1371/journal.pone.0246504 (PMC7935316; doi:10.1371/journal.pone.0246504)
Supplement: S1 Fig — (A), (B), (C) Percentage of fish that recovered by age for concentrations of 0.008%, 0.009% and 0.01% MS-222 respectively. Black lines represent the 90% confidence interval for each value, calculated using the sample size (see materials and methods). (PDF) [file pone.0246504.s001.pdf]

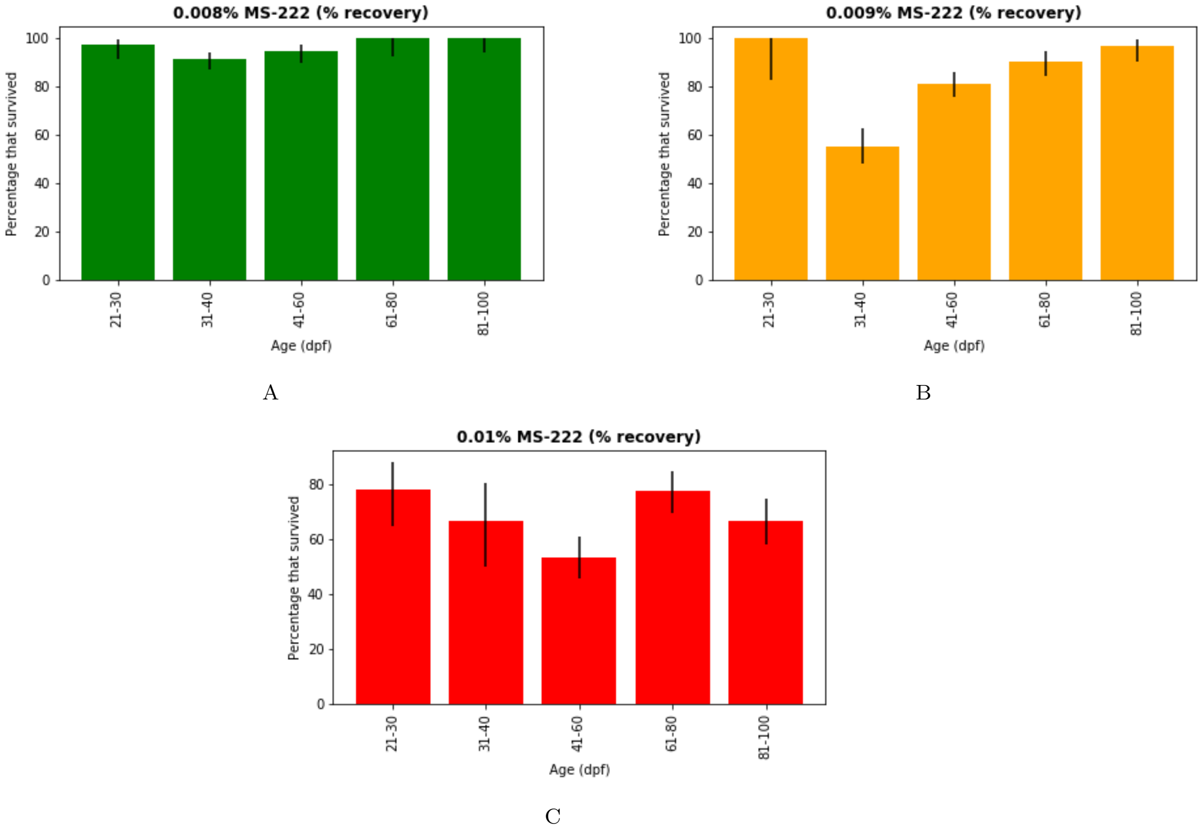

Supplementary Fig. 1: Age is not a good predictor of recovery. (A), (B), (C) Percentage of fish that recovered by age for concentrations of 0.008%, 0.009% and 0.01% MS-222 respectively. Black lines represent the 90% confidence interval for each value, calculated using the sample size (see materials and methods).
