# Supplementary material for: A suitable anaesthetic protocol for metamorphic zebrafish
Source: PLoS One. 2021 Mar 5;16(3):e0246504. doi: 10.1371/journal.pone.0246504 (PMC7935316; doi:10.1371/journal.pone.0246504)
Supplement: S4 Fig — Boxplot of (A) induction time, (B) time taken to lose touch responsivity, (C) heartbeats per minute at 5:00, (D) visible breaths per minute at 5:00, (D) time taken to regain movement and (E) time taken to fully recover against repeat number when using Protocol 4 to repeatedly anaesthetise and recover fish. Stars indicate significant difference as determined using the Mann-Whitney U test. ns = not significant, ‘*’ indicates p<0.05, ‘**’ indicates p<0.01, ‘***’ indicates p < 0.001 and ‘****’ indicates p<0.0001. CP stands for cotton pad maintenance method. (PDF) [file pone.0246504.s004.pdf]

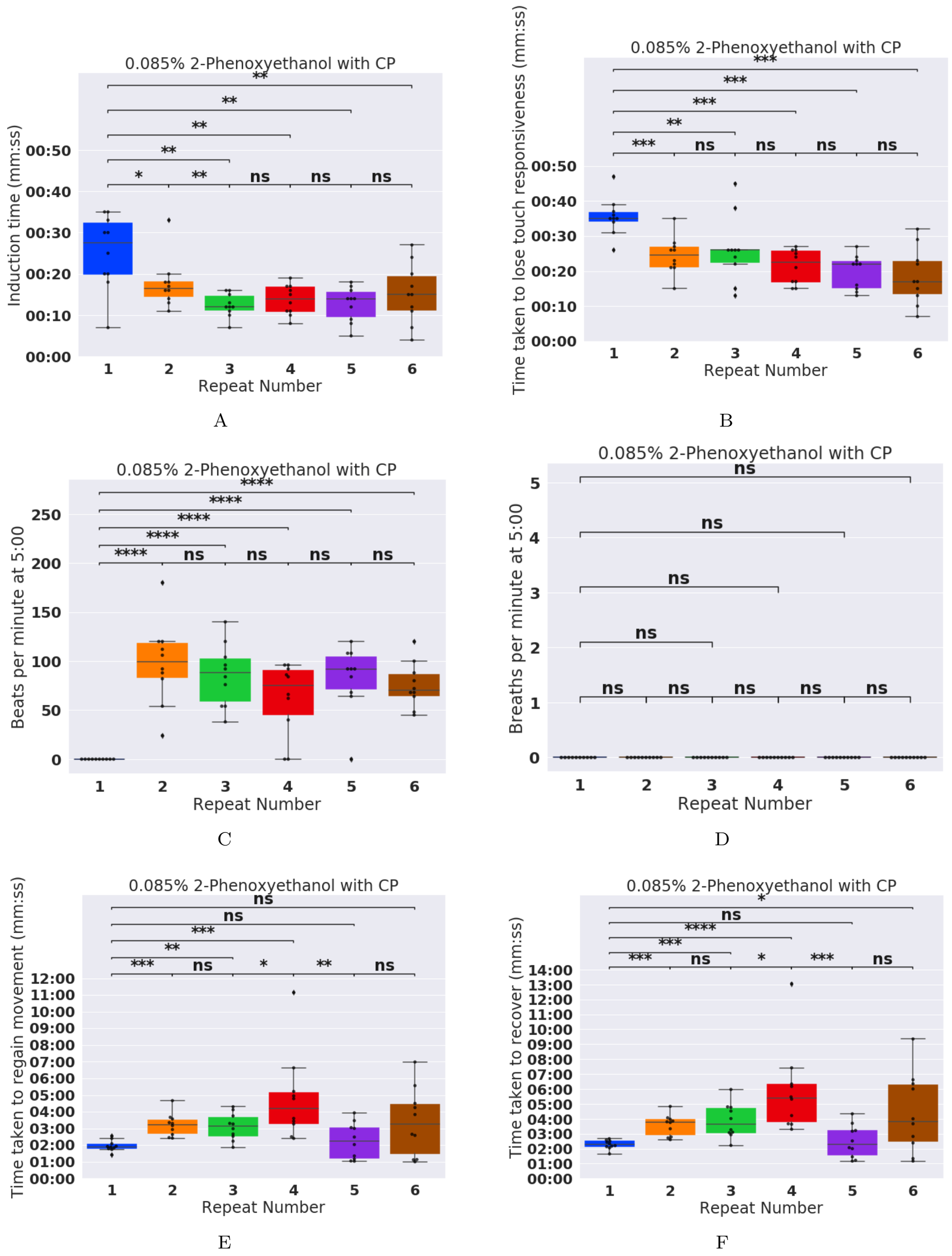

Supplementary Fig. 4: The time taken to induce, lose touch responsiveness, regain movement, recover as well as respiratory rate varies with repeat number when repeatedly anaesthetised using protocol 4 every 4 days. Boxplot of (A) induction time, (B) time taken to lose touch responsivity, (C) heartbeats per minute at 5:00, (D) visible breaths per minute at 5:00, (D) time taken to regain movement and (E) time taken to fully recover against repeat number when using Protocol 4 to repeatedly anaesthetise and recover fish. Stars indicate significant difference as determined using the Mann-Whitney U test. ns = not significant, '*' indicates $p<0.05$, '**' indicates $p<0.01$, '***' indicates $p < 0.001$ and '****' indicates $p<0.0001$. CP stands for cotton pad maintenance method.
