# Supplementary material for: A suitable anaesthetic protocol for metamorphic zebrafish
Source: PLoS One. 2021 Mar 5;16(3):e0246504. doi: 10.1371/journal.pone.0246504 (PMC7935316; doi:10.1371/journal.pone.0246504)
Supplement: S7 Fig — Scatter plot of (A) induction time, (B) time taken to lose touch responsivity, (C) beats per minute at 5:00, (D) breaths per minute at 5:00, (E) time taken to regain movement and (F) time taken to fully recover against repeat number over their standard length (mm). Blue dots correspond to fish that were repeatedly dosed. Red dots correspond to fish that were dosed once (i.e. control group). Blue and red linear regression lines are superimposed with translucent 95% confidence interval. (PDF) [file pone.0246504.s007.pdf]

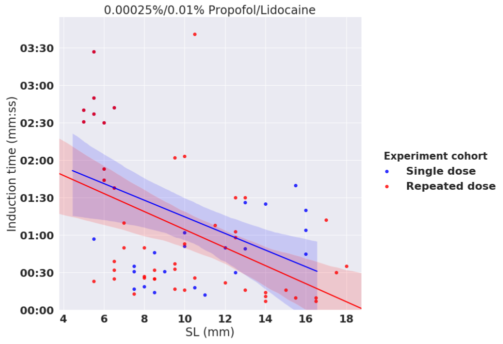

A

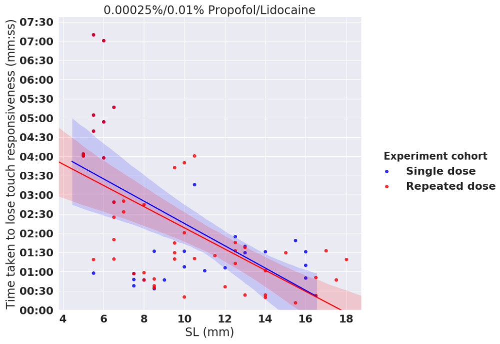

B

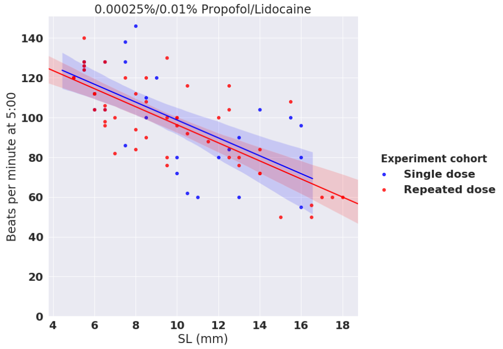

C

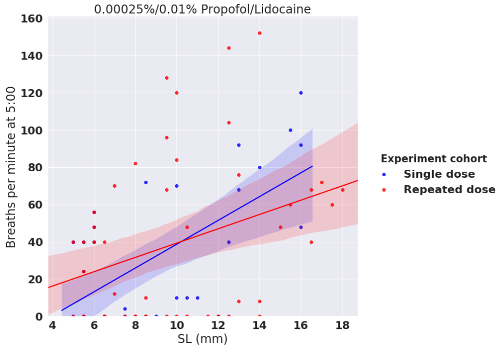

D

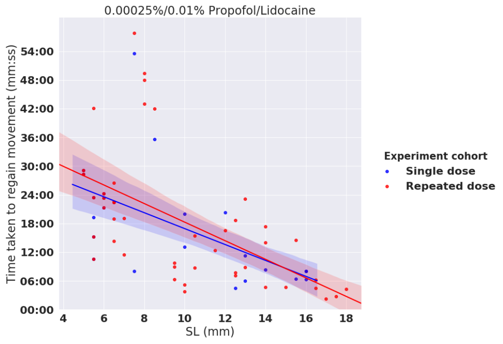

E

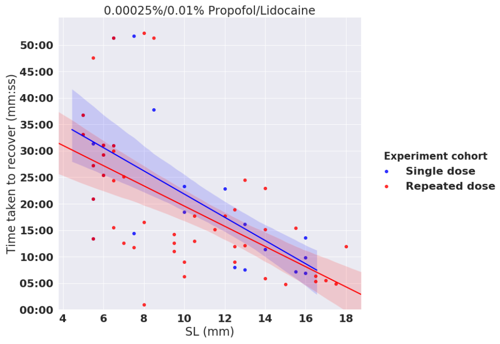

F

Supplementary Fig. 7: The time taken to induce, lose touch responsiveness, regain movement, recover as well as respiratory rate varies with SL when repeatedly anaesthetised using protocol 5 every 4 days. Scatter plot of (A) induction time, (B) time taken to lose touch responsivity, (C) beats per minute at 5:00, (D) breaths per minute at 5:00, (E) time taken to regain movement and (F) time taken to fully recover against repeat number over their standard length (mm). Blue dots correspond to fish that were repeatedly dosed. Red dots correspond to fish that were dosed once (*i.e.* control group). Blue and red linear regression lines are superimposed with translucent 95% confidence interval.
