# Supplementary material for: A suitable anaesthetic protocol for metamorphic zebrafish
Source: PLoS One. 2021 Mar 5;16(3):e0246504. doi: 10.1371/journal.pone.0246504 (PMC7935316; doi:10.1371/journal.pone.0246504)
Supplement: S5 Table — Multiple linear regression model for y = induction time, time taken to lose touch responsiveness, beats per minute, breaths per minute, time taken to regain movement and time taken to recover under protocol 4 when used every 2 days. For each y value a significant regression equation was found if p<0.05 and can be described as (F(Df model, Df residual) = F-stat. The predicted y value is given by y = C+m1*(repeat number)+m2*(standard length), where repeat number is the number of doses the fish will have been exposed to at the end of the experiment and standard length is in mm. Values highlighted are those where the associated p-value is <0.05 and thus can be deemed as significant. (DOCX) [file pone.0246504.s014.docx]

**S5 Table:** **The effects of repeat number and SL on success measures for protocol 4 when used every 2 days.** Multiple linear regression model for y= induction time, time taken to lose touch responsiveness, beats per minute, breaths per minute, time taken to regain movement and time taken to recover under protocol 4 when used every 2 days. For each y value a significant regression equation was found if p<0.05 and can be described as (F(Df model, Df residual)=F-stat. The predicted y value is given by y=C+m_1_*(repeat number)+m_2_*(standard length), where repeat number is the number of doses the fish will have been exposed to at the end of the experiment and standard length is in mm. Values highlighted are those where the associated p-value is <0.05 and thus can be deemed as significant.

| **Protocol 4 (Every 2 days)** | **Df res.** | **Df Model** | **F-stat** | **p** | **R^2^** | **C** | **Repeat number coef. (m_1_)** | **Repeat number p-value** | **SL coef.**  **(m_2_)** | **SL**  **p-value** |
| --- | --- | --- | --- | --- | --- | --- | --- | --- | --- | --- |
| **Induction time (s)** | 87 | 2 | 7.89 | <0.001 | 0.154 | 14.4 | -4.666 | <0.05 | 2.69 | <0.05 |
| **Time taken to lose touch responsiveness (s)** | 87 | 2 | 16.26 | <0.001 | 0.272 | 29.26 | -6.25 | <0.05 | 2.96 | <0.05 |
| **Time taken to regain movement (s)** | 87 | 2 | 17.24 | <0.001 | 0.284 | 211.3 | -8.48 | 0.07 | -4.61 | 0.18 |
| **Time taken to recover (s)** | 87 | 2 | 14.39 | <0.001 | 0.249 | 232.4 | -12.86 | 0.02 | -1.98 | 0.62 |
